# Tribological Properties of Plasma-Based Low-Energy Nitrogen Ion Implanted 17-4PH Martensitic Stainless Steel

**DOI:** 10.3390/ma19050887

**Published:** 2026-02-27

**Authors:** Xu Yang, Honglong Che, Shuyuan Li, Mingkai Lei

**Affiliations:** Surface Engineering Laboratory, School of Materials Science and Engineering, Dalian University of Technology, Dalian 116024, China; yx0727@mail.dlut.edu.cn (X.Y.); chehl@dlut.edu.cn (H.C.); syli@mail.dlut.edu.cn (S.L.)

**Keywords:** 17-4PH martensitic stainless steel, plasma-based low-energy nitrogen ion implantation, nanostructure, tribological properties, wear mechanism

## Abstract

**Highlights:**

**What are the main findings?**

- Nitrided layer thickness, surface nitrogen concentration, and hardness increase with temperature.
- Highest wear resistance is achieved at 450 °C with nanocrystalline γ′_N_ and minor CrN.
- The nanostructure combined with limited CrN precipitation enhances both hardness and toughness, improving resistance to plastic deformation.
- The wear mechanism undergoes a transition from adhesive to oxidative and fatigue after nitriding process.

**What are the implication of the main findings?**

- Nitriding significantly extends the service life of stainless steel under dry sliding conditions.

**Abstract:**

This study investigates the tribological properties of 17-4PH martensitic stainless steel modified by plasma-based low-energy nitrogen ion implantation to enhance its surface hardness and wear resistance. The steel was nitrided at temperatures of 350 °C, 450 °C, and 550 °C for 4 h, and the resultant layers were characterized with respect to microstructure, hardness, and composition. Tribological tests were performed using a ball-on-disk tribometer under dry sliding conditions against an Si_3_N_4_ ceramic ball, with normal loads of 2–8 N and sliding speeds of 0.15–0.60 m/s. The results demonstrate that the nitrided layer thickness increased from 11 μm to 27 μm and the surface nitrogen concentration rose from 29.7 at.% to 33.1 at.% with increasing temperature, accompanied by an increase in nanocrystallite size from 2 nm to 15 nm and enhanced hardness from 13.51 GPa to 15.66 GPa. All nitrided layers exhibited significantly improved wear resistance relative to the unmodified steel. The layer nitrided at 450 °C demonstrated optimal performance due to a refined nanostructure and minor CrN that enhance plastic deformation resistance and facilitate oxide film formation. While, the 350 °C treated layer exhibits diminished thickness and reduced hardness, and the 550 °C treatment induces excessive CrN precipitation and micro-cracking, consequently compromising both toughness and wear resistance.

## 1. Introduction

As a precipitation-hardening martensitic stainless steel, 17-4PH combines superior mechanical properties, excellent corrosion resistance, and amenability to precipitation hardening [1,2,3]. Consequently, it finds extensive application in advanced equipment manufacturing sectors such as nuclear power, aerospace, and petrochemical engineering, serving as a critical material for key friction pair components including bearings, gears, and valve cores [4,5]. However, its relatively low surface hardness predisposes it to severe wear failure under prolonged service. Enhancing the surface hardness and wear resistance of 17-4PH martensitic stainless steel through surface modification, while preserving its inherent corrosion resistance, constitutes a crucial technical objective for extending service life.

Plasma nitriding, carburizing, and nitrocarburizing represent effective surface modification techniques for 17-4PH. These processes enhance surface hardness and wear resistance by forming a hardened modified layer [6,7,8], while the relatively low treatment temperatures minimize detrimental effects on the substrate’s mechanical properties. The resultant surface hardness and wear resistance are intrinsically linked to the phase composition of the modified layers. Li et al. [9] reported that nitriding below 420 °C primarily produces nitrogen-expanded martensite (α’_N_) with minor nitrogen expanded austenite (γ_N_), yielding a surface hardness approximately double that of the unmodified stainless steel. Above 420 °C, a mixed phase structure emerges, comprising γ’-Fe_4_N-like ordered nitrogen-expanded austenite (γ’_N_), hexagonal close-packed ε-Fe_3_N nitride, and CrN precipitates [10,11]. Consequently, the wear loss of the modified layer decreases by approximately an order of magnitude. Under unlubricated pin-on-disk testing against a WC ball, the dominant wear mechanism shifts from the adhesive and abrasive wear observed in the unmodified stainless steel to mild oxidative wear for the modified layer [12]. Furthermore, the nanostructure of the modified layer significantly influences wear behavior and underlying mechanisms. Liu et al. [13] found that modified layers formed at 400–500 °C, composed of nanocrystalline γ’-Fe_4_N, α’_N_, and CrN with grain sizes between 5 and 200 nm, exhibited a 1.5-fold increase in surface hardness and a two-order-of-magnitude reduction in wear rate. Under similar sliding conditions, slight abrasive wear dominated the mechanism. Despite these advances, investigations into the evolution of wear mechanisms for nitrided layers under complex conditions—such as variable normal load, variable sliding speed, or their coupling effects—remain insufficient.

In this study, the tribological properties of 17-4PH martensitic stainless steel modified by plasma-based low-energy nitrogen ion implantation at the temperature ranging from 350 °C to 550 °C for 4 h were systematically investigated on a ball-on-disk tribometer under unlubricated conditions against an Si_3_N_4_ ceramic ball counterface. The correlation between the microstructures of nitrided layers at different temperatures and wear behaviors of the 17-4PH martensitic stainless steel was systematically studied under the different normal loads and sliding speeds conditions.

## 2. Experimental

The 17-4PH stainless steel specimens, possessing the chemical composition (in wt.%) of 0.07C, 0.40Si, 0.01S, 0.02P, 0.32Nb, 16.03Cr, 4.46Ni, 4.25Cu, 0.82Mn, and balance Fe, were utilized for nitriding. The two-stage pre-nitriding heat treatment process was performed to obtain a uniform martensitic microstructure and to relieve internal stresses. Initially, quenching was conducted at 1040 °C for 2 h, followed by oil cooling. Subsequently, annealing was performed at 595 °C for 4 h. These after-treated samples were defined as unmodified stainless steel for the following nitriding. Cylindrical samples measuring 17 mm in diameter and 5 mm in thickness were sectioned from the heat-treated stainless steel. Prior to nitriding, all samples were ground and polished according to standard metallographic procedures, ultrasonically cleaned in acetone for 600 s, and air-dried.

Plasma nitriding was performed using a plasma-based low-energy ion implantation system equipped with a high-density, high-ionization-degree electron cyclotron resonance (ECR) microwave plasma source. The nitriding parameters were listed in Table 1. The chamber was initially evacuated to a base pressure of 1.5 × 10^−3^ Pa [14]. High-purity nitrogen gas was introduced to maintain a process pressure of 5 × 10^−2^ Pa throughout nitriding. A pulsed negative bias voltage of −2 kV, with a repetition rate of 1000 Hz and a pulse width of 250 μs, was applied to the sample holder. A stable plasma was generated at a microwave power of 200 W. Nitriding treatments were conducted at three distinct temperatures: 350 °C, 450 °C, and 550 °C for 4 h each. An auxiliary heater integrated within the sample holder regulated the nitriding temperature. Sample temperature was monitored in situ during processing using a thermocouple. An average nitrogen ion current density of 0.8 mA/cm^2^ was applied to ensure a high implantation dose of low-energy nitrogen ions. The plasma density ranged from approximately 5 × 10^11^ cm^−3^ to 1.5 × 10^12^ cm^−3^. Following nitriding, samples underwent furnace cooling to room temperature under a continuous N_2_ gas flow for subsequent microstructural characterization and tribological testing.

The cross-sections of the nitrided layers were prepared by progressive grinding, polishing, and etching. The etchant consisted of a solution containing 10 g of analytical grade FeCl_3_, 10 mL of analytical grade HCl, and 120 mL of deionized water. The morphology and thickness of the nitrided layer were examined using a light optical microscopy (LOM, DMi8, Leica Microsystems, Wetzlar, Germany). Nitrogen concentration-depth profiles within the nitrided layers were determined using a field emission electron probe microanalyzer (EPMA, JXA-8530F PLUS, JEOL Ltd., Tokyo, Japan) operated at an accelerating voltage of 30 kV. Phase constituents were analyzed by a X-ray diffraction (XRD, Empyrean, Malvern Panalytical Ltd., Almelo, The Netherlands) utilizing CuKα radiation with λ = 0.15405 nm over a 2θ scanning range of 20° to 100°. The microstructure of the nitrided layer was characterized by dark-field (DF) imaging and selected area electron diffraction (SAED) patterns using a transmission electron microscope (TEM JEM-F200, JEOL Ltd., Tokyo, Japan) operated at 200 kV. The grain size was evaluated by Nano Measurer (version 1.2). Thin foils for TEM analysis were prepared from the top-surface of the nitrided layers using a dual-beam focused ion beam (FIB, Helios G4 UX, FEI Company, Hillsboro, OR, USA) with Ga^+^ ions for thinning. A protective Pt layer was deposited on the surface prior to the lift-out and thinning procedures to shield the underlying nitrided layer from Ga^+^ ion beam damage during subsequent FIB milling.

The hardness of the nitrided layer surface and cross-section was measured using an Vickers microhardness tester (HXD-1000TM, SOIF, Shanghai, China) under a 0.25 N load. Tribological tests were conducted on a tribometer (WTM-2E, ZHONGKEKAIHUA Ltd., Lanzhou, China) in a ball-on-disk configuration under dry sliding conditions, wherein the surface of a stationary top-mounted ball was rubbed against a reciprocating flat sample. The counterface material was a 4 mm-diameter Si_3_N_4_ ceramic ball, with applied normal loads of 2 N and 8 N. The wear track diameter was 10 mm. The friction coefficient was recorded during wear testing via a transducer mounted on the tribometer’s load arm. For each tribological test, a fresh ball-disk assembly was slid at relative sliding speeds of 0.15 m/s and 0.60 m/s for a duration of 7200 s. Cross-sectional profiles of the wear track were measured using a profilometer (Surfcorder ET 4000M, Kosaka Laboratory Ltd., Tokyo, Japan) equipped with a 2 μm-radius pinhead. The wear volume *V* of the wear track was calculated by the following equation:

(1) V=π×d×S,

where *d* represents the rotating diameter, and *S* denotes the integrated area of the two-dimensional wear track profile obtained using Origin Pro 8.6.0 software. The specific wear rate *K* was determined using the following equation:

(2) $$K=\frac{V}{P\times L},$$

where *P* denotes the normal load, and *L* represents the total sliding distance, defined as the product of sliding speed and test duration. The worn surface morphology and elemental distribution of the sample were examined using a scanning electron microscope (SEM, Zeiss SUPRA55, Oberkochen, Germany) equipped with an energy dispersive spectrometer (EDS).

## 3. Results

### 3.1. Microstructure of the Nitrided Layer

**Figure 1:**
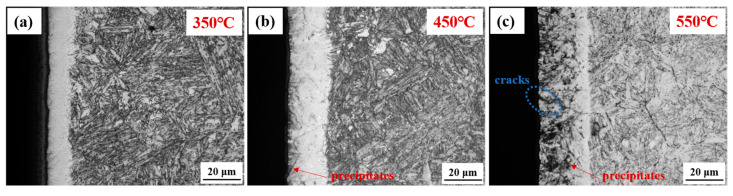
Cross-sectional optical micrographs of the nitrided 17-4PH martensitic stainless steel at (**a**) 350 °C, (**b**) 450 °C, and (**c**) 550 °C for 4 h [15].

Figure 1 presents cross-sectional optical micrographs of the nitrided 17-4PH martensitic stainless steel processed at temperatures of 350 °C, 450 °C and 550 °C. The matrix beneath the nitrided layers exhibited a microstructure similar to the base material, characterized by lath martensite (α’) and a minor amount of residual austenite (γ_re_). At 350 °C, a continuous white layer with an average thickness of approximately 11 μm formed on the steel surface. The average thickness of the nitrided layer progressively increased with rising nitriding temperature, reaching approximately 21 μm at 450 °C and further increasing to about 27 μm at 550 °C. Furthermore, precipitates were first detected along grain boundaries at 450 °C. Both the volume fraction and the depth of these precipitates increased with higher nitriding temperatures. At 550 °C, the micro-cracks were observed within the nitrided layer, as marked in Figure 1c.

**Figure 2:**
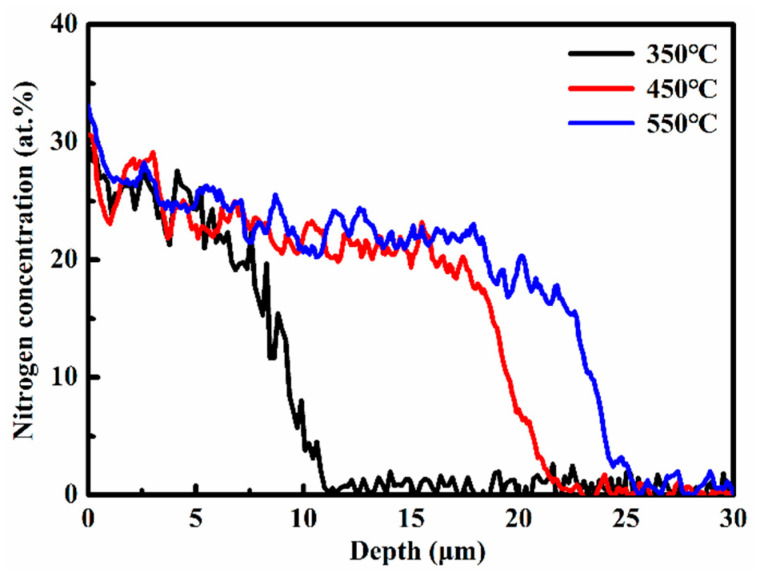
Nitrogen concentration-depth profiles of the nitrided 17-4PH martensitic stainless steel at 350–550 °C for 4 h [15].

Figure 2 presents the nitrogen concentration-depth profiles of nitrided 17-4PH martensitic stainless steel treated at temperatures of 350 °C, 450 °C and 550 °C. At 350 °C, the maximum surface nitrogen concentration of the nitrided layer reaches approximately 29.7 at.%. The surface nitrogen concentration exhibits a progressive increase with elevating nitriding temperature. At 450 °C, this value rises to about 30.6 at.%, and further attains 33.1 at.% at 550 °C. This temperature-dependent enhancement in surface nitrogen concentration is ascribed to the accelerated inward diffusion of nitrogen atoms at higher nitriding temperatures. All profiles exhibit a characteristic nitrogen concentration plateau, with nitrogen penetration depths extending from approximately 11 μm to 26 μm across the investigated temperature range of 350–550 °C. This plateau formation correlates with the trapping and detrapping of interstitial nitrogen atoms by chromium atoms in solid solution [16]. Furthermore, pronounced nitrogen concentration gradients manifest as steep profiles within the transition zone between the nitrided layer and the substrate in all specimens.

**Figure 3:**
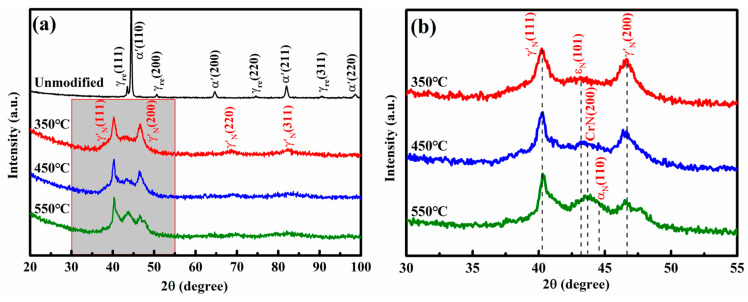
XRD patterns of the unmodified 17-4 PH specimen after quenching and annealing and the nitrided 17-4PH martensitic stainless steel at 350–550 °C for 4 h. (**a**) Full scaling pattern from 20 to 100°; (**b**) local view enlarged in (**a**) from 30 to 55° [15].

Figure 3 presents the XRD patterns of 17-4PH martensitic stainless steel after nitriding at temperatures of 350 °C, 450 °C and 550 °C. The unmodified stainless steel consists of martensite (α’) and retained austenite (γ_re_). After nitriding, all diffractograms exhibit peaks corresponding to nitrogen expanded austenite (γ’_N_) with a γ′-Fe_4_N-type structure and the hexagonal close-packed phase (ε_N_). Compared with the γ_re_ reflections, the γ’_N_ peaks are shifted toward lower 2θ angles, which is attributed to the lattice expansion caused by the interstitial dissolution of nitrogen in the face-centered cubic matrix. The observed peak broadening primarily results from grain refinement or the high density of defects [17]. As the nitriding temperature rises, these peaks become sharper, indicating coarsening of the grains. A diffraction peak near 44° can be assigned to CrN. Its intensity increases with nitriding temperature, reflecting a higher volume fraction of CrN precipitates, as is consistent with the observation in Figure 1c. At 550 °C, an additional peak associated with nitrogen-containing ferrite α_N_ emerges, suggesting the onset of γ’_N_ decomposition.

**Figure 4:**
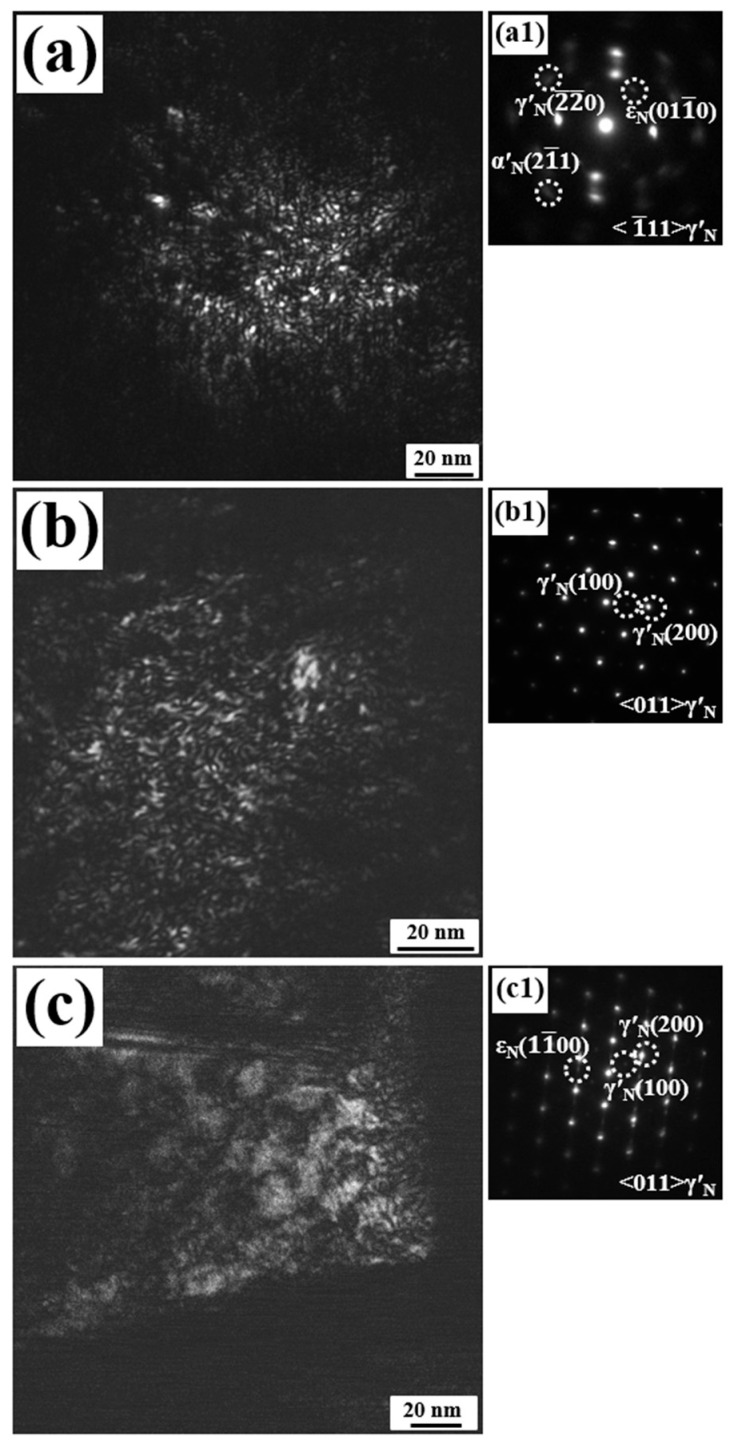
DF-TEM images and the corresponding SAED patterns of the nitrided 17-4PH martensitic stainless steel at (**a**) 350 °C, (**b**) 450 °C, and (**c**) 550 °C for 4 h. (**a1**–**c1**) The corresponding SAED patterns [15].

Figure 4 presents dark-field TEM (DF-TEM) images and the corresponding selected-area electron diffraction (SAED) patterns of 17-4PH martensitic stainless steel nitrided at 350 °C, 450 °C and 550 °C. The SAED patterns in Figure 4 (a1–c1) correspond directly to the respective DF-TEM micrographs. Across the entire temperature range studied, the nitrided layer consistently exhibited a nanocrystalline structure, confirming that low-temperature plasma nitriding induces nanocrystallization in this stainless steel. The nanocrystallization is driven by the coupling of chemical and elastic diffusion fields at a low temperature, which triggers a self-sustaining periodic diffusionless austenitic transformation in nanoscale, resulting in sequential layer-by-layer nanocrystallization, which is introduced in another work in more detail [18]. The average grain size increased gradually with nitriding temperature: from approximately 2 nm at 350 °C, to about roughly 15 nm at 550 °C, because of higher mobility for metal atoms. This trend agrees well with the XRD results.

### 3.2. Hardness of the Nitrided Layer

**Figure 5:**
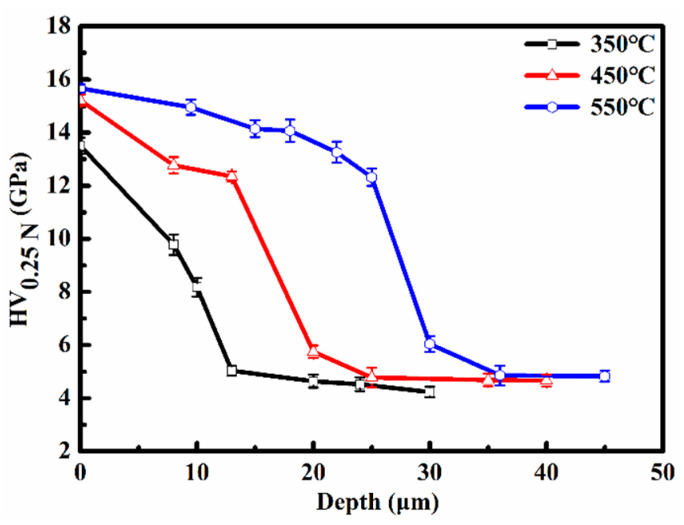
Hardness-depth profiles of the nitrided 17-4PH martensitic stainless steel at 350–550 °C for 4 h.

Figure 5 illustrates the hardness-depth profiles of the nitrided 17-4PH martensitic stainless steel subjected to nitriding at temperatures ranging from 350 °C to 550 °C. At 350 °C, the maximum surface hardness is HV_0.25N_ 13.51 GPa, approximately three times higher than that of the unmodified stainless steel with HV_0.25N_ 3.05 GPa. The hardness gradually decreases to a plateau before dropping sharply to the matrix at a depth of about 13 μm. This hardness-depth profile closely follows the nitrogen concentration-depth distribution. A similar trend in hardness variation is observed across the nitriding temperature range of 350–550 °C. The surface hardness gradually increases with increasing nitriding temperature. At 550 °C, the surface hardness reaches approximately HV_0.25N_ 15.66 GPa. The enhanced hardness of the nitrided layers primarily arises from solid solution strengthening due to interstitial nitrogen atoms [19]. Furthermore, the formation of the nanocrystalline microstructure results in enhanced surface hardness [13].

### 3.3. Tribological Properties of the Nitrided Layer

**Figure 6:**
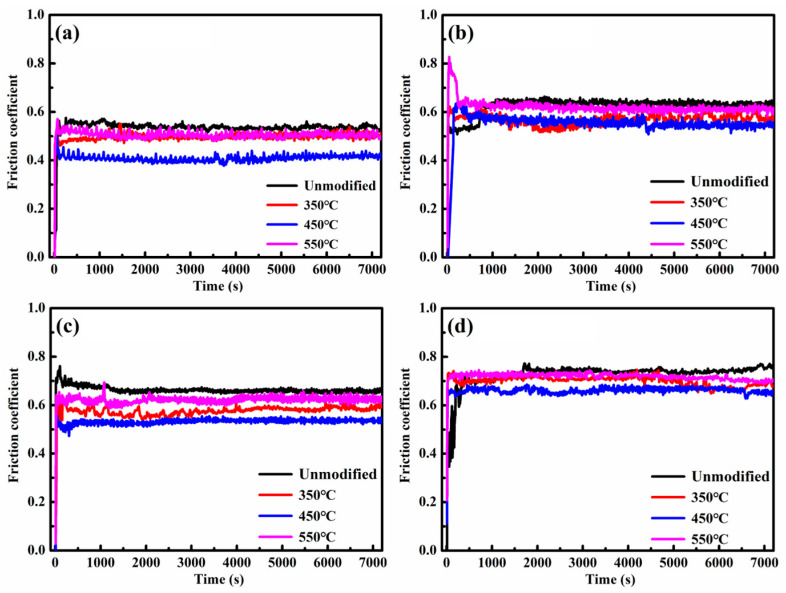
Friction coefficient curves relative to sliding time for the unmodified and nitrided 17-4PH martensitic stainless steel against Si_3_N_4_ ball counterface under the normal load and the sliding speed of (**a**) 2 N/0.15 m/s, (**b**) 2 N/0.60 m/s, (**c**) 8 N/0.15 m/s and (**d**) 8 N/0.60 m/s.

Figure 6 illustrates the friction coefficient as a function of sliding time for unmodified and nitrided 17-4PH martensitic stainless steel. Specimens underwent nitriding at temperatures of 350, 450, and 550 °C, followed by tribological testing against a Si_3_N_4_ ball counterface under sliding speeds of 0.15 and 0.60 m/s and normal loads of 2 N and 8 N. Under identical wear conditions, both unmodified and nitrided specimens exhibited comparable evolution patterns in friction behavior. However, the friction coefficients measured for the nitrided layers were significantly lower than those of the unmodified substrate. Notably, the layer nitrided at 450 °C exhibited the lowest friction coefficient. Both materials demonstrated a marked elevation in friction coefficients with increasing normal load and/or sliding speed.

Under a normal load of 2 N and a sliding speed of 0.15 m/s, the friction coefficients for both unmodified and nitrided stainless steel increased rapidly upon sliding initiation, stabilizing within approximately 100 s to 200 s. The stable friction coefficient for the 450 °C nitrided layer was approximately 0.41, lower than the value of 0.53 observed for the unmodified steel. Increasing the sliding speed to 0.60 m/s elevated the friction coefficient of the 450 °C nitrided layer to approximately 0.54, which remained consistently lower than the 0.64 recorded for the unmodified stainless steel. Friction coefficients exhibited heightened instability during testing at this elevated sliding speed of 0.60 m/s.

Under a higher normal load of 8 N and a low sliding speed of 0.15 m/s, the 450 °C nitrided layer demonstrated a friction coefficient of 0.54, again lower than the value of 0.66 for the unmodified stainless steel. Friction behavior remained unstable throughout testing under this increased load of 8 N. Further increasing the sliding speed to 0.60 m/s elevated the friction coefficients to 0.74 and 0.67 for the unmodified stainless steel and the 450 °C nitrided layer, respectively.

In summary, the nitrided 17-4PH stainless steel consistently exhibits lower and more stable friction coefficients compared to the unmodified steel, with the lowest reduction achieved at 450 °C.

**Figure 7:**
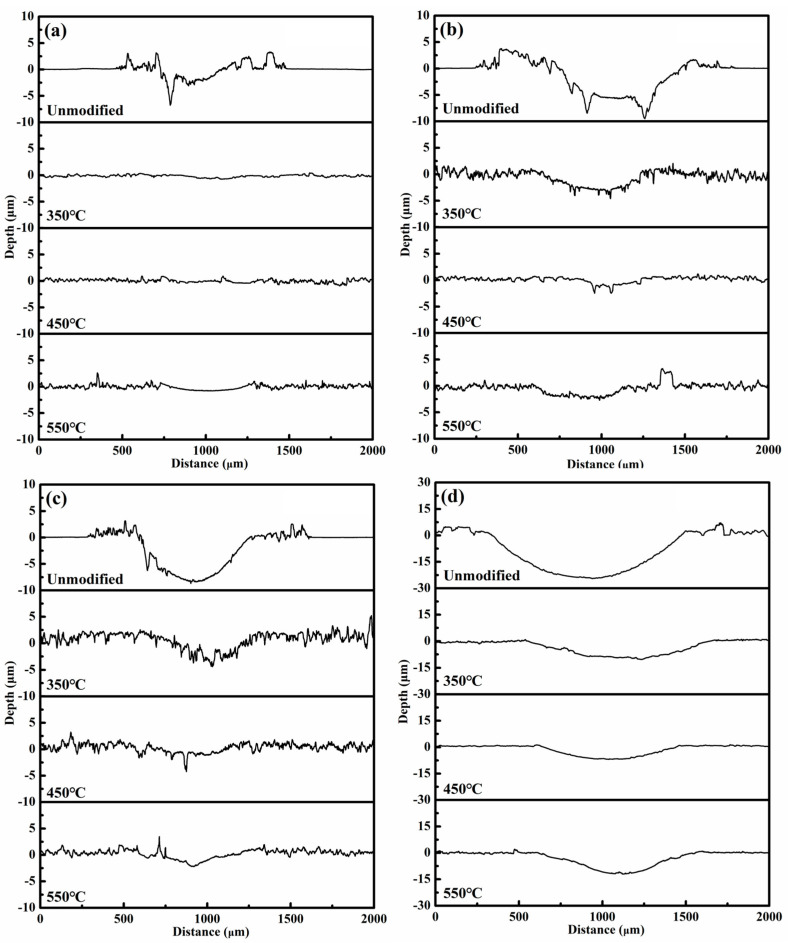
Cross-sectional profilometer pattern of wear tracks for the unmodified and nitrided 17-4PH martensitic stainless steel against Si_3_N_4_ ball counterface under the normal load and the sliding speed of (**a**) 2 N/0.15 m/s, (**b**) 2 N/0.60 m/s, (**c**) 8 N/0.15 m/s and (**d**) 8 N/0.60 m/s.

Figure 7 presents the cross-sectional profilometry profiles of wear tracks for unmodified and nitrided 17-4PH martensitic stainless steel at the temperatures of 350 °C, 450 °C, and 550 °C. Wear testing was performed against a Si_3_N_4_ ball counterface under sliding speeds of 0.15 m/s and 0.6 m/s, and normal loads of 2 N to 8 N. The unmodified 17-4PH martensitic stainless steel exhibits material accumulation (built-up lips) at the wear track edges. Conversely, the wear tracks on the nitrided layers exhibit relatively flat profiles. The width and depth of the parallel grooves within the wear tracks, oriented along the sliding direction, increase significantly with rising sliding speed from 0.15 m/s to 0.6 m/s and normal load from 2 N to 8 N. The widest and deepest grooves occur under the highest tested sliding speed of 0.6 m/s and normal load of 8 N. Under identical wear conditions, the width and depth of wear tracks on the nitrided layers at 350–550 °C are significantly reduced compared to those on the unmodified stainless steel. At 450 °C, the nitrided layers demonstrate wear tracks with notably reduced width and depth.

**Figure 8:**
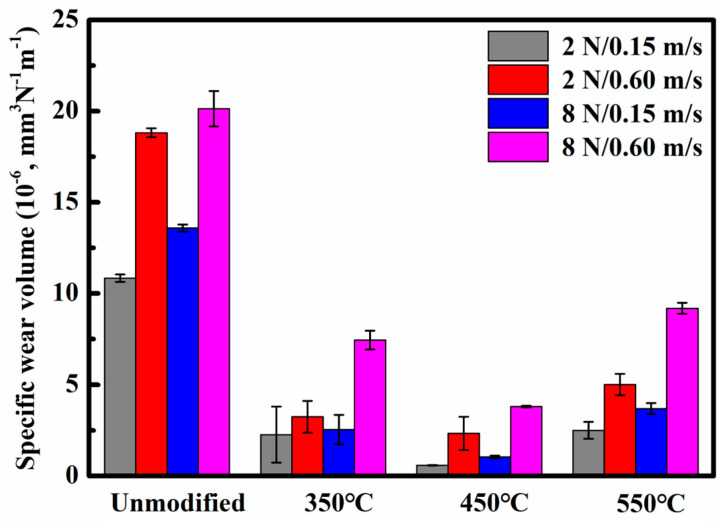
Specific wear rate of the unmodified and nitrided 17-4PH martensitic stainless steel against Si_3_N_4_ ball counterface under the sliding speeds of 0.15–0.6 m/s and the normal loads of 2–8 N.

Figure 8 presents the wear rate data of unmodified and nitrided 17-4PH martensitic stainless steel at 350 °C, 450 °C, and 550 °C tribological testing with Si_3_N_4_ balls. Results indicate that the specific wear rate of 17-4PH stainless steel increased significantly with both sliding speed and normal load. Under the highest sliding speed of 0.60 m/s and maximum normal load of 8 N, all specimens exhibited the highest wear volume and specific wear rate. Under identical wear conditions, the nitrided layers at 350 °C, 450 °C, and 550 °C exhibited significantly lower specific wear rates compared to unmodified stainless steel, demonstrating that nitriding effectively enhances surface wear resistance. Notably, the nitrided layer at 450 °C demonstrated the most outstanding wear resistance, with its specific wear rate being the lowest among all test groups. Specific data indicate that under a low load of 2 N and a low sliding speed of 0.15 m/s conditions, the specific wear rate of the nitrided layer at 450 °C was only 0.58 × 10^−6^ mm^3^ N^−1^m^−1^, significantly lower than that of the unmodified stainless steel of 10.84 × 10^−6^ mm^3^ N^−1^m^−1^. When the normal load increased to 8 N and the sliding speed rose to 0.60 m/s, the specific wear rate of the nitrided layer at 450 °C reaches 3.80 × 10^−6^ mm^3^ N^−1^m^−1^, yet is still significantly lower than that of the unmodified stainless steel with 20.13 × 10^−6^ mm^3^ N^−1^ m^−1^ under the same conditions. In summary, nitriding treatment within the 350–550 °C range significantly improves the wear resistance of 17-4PH martensitic stainless steel, with the nitrided layer at 450 °C achieving the best surface layer performance and lowest specific wear rates.

**Figure 9:**
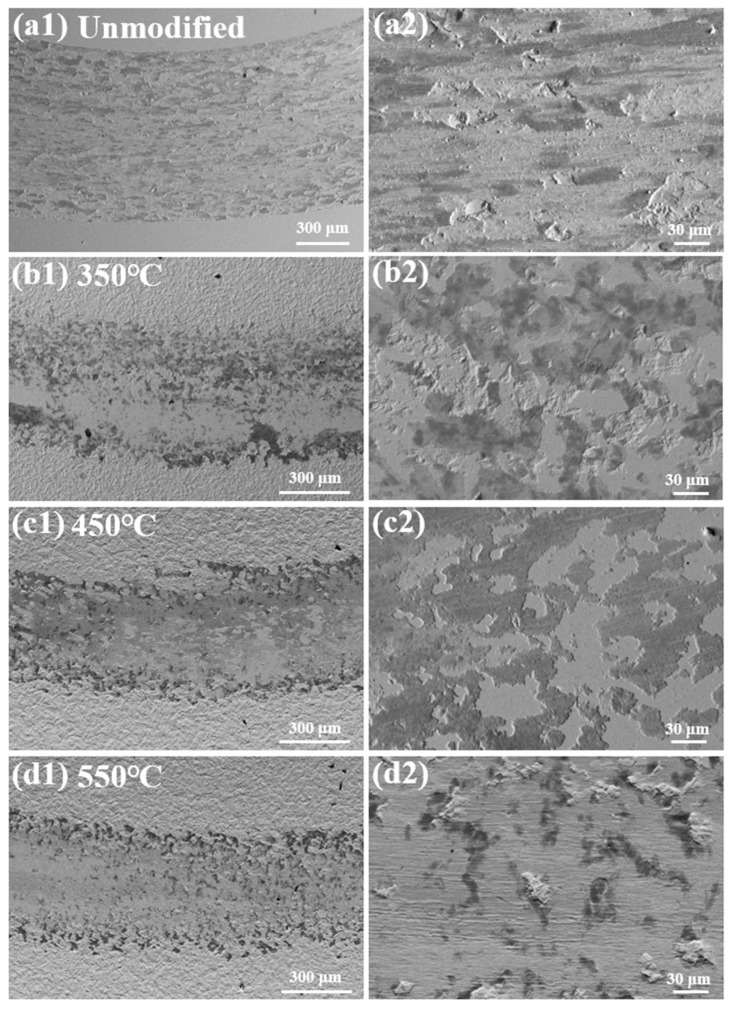
Worn surface morphologies of the unmodified and nitrided 17-4PH martensitic stainless steel at 350–550 °C against Si_3_N_4_ ball counterface under the sliding speed of 0.15 m/s and the normal load of 2 N. (**a1**,**a2**) Unmodified, (**b1**,**b2**) 350 °C, (**c1**,**c2**) 450 °C and (**d1**,**d2**) 550 °C.

Figure 9 shows the worn surface morphologies of the unmodified and nitrided 17-4PH martensitic stainless steel at 350–550 °C against Si_3_N_4_ ball counterface under the sliding speed of 0.15 m/s and the normal load of 2 N. Abrasion traces on unmodified martensitic stainless steel exhibit roughness characterized by shallow furrows and discrete fine spalled debris, indicating a mild adhesive wear mechanism. Conversely, specimens nitrided at 350–550 °C demonstrate significantly smoother wear tracks. At 350 °C, the worn surface is characterized by interconnected flaky spalls and fragmented oxide films, signifying a transition to oxidative wear mechanism. When the nitriding temperature increases to 450 °C, the wear track becomes even smoother, featuring smeared oxide films covering localized surface regions. Under these test conditions, oxidative wear predominates the wear behavior. At 550 °C, the worn surface presents fewer oxide films accompanied by abundant fragmented debris and slight plastic deformation. These morphological features correspond to an oxidative wear and fatigue wear mechanism [20].

**Figure 10:**
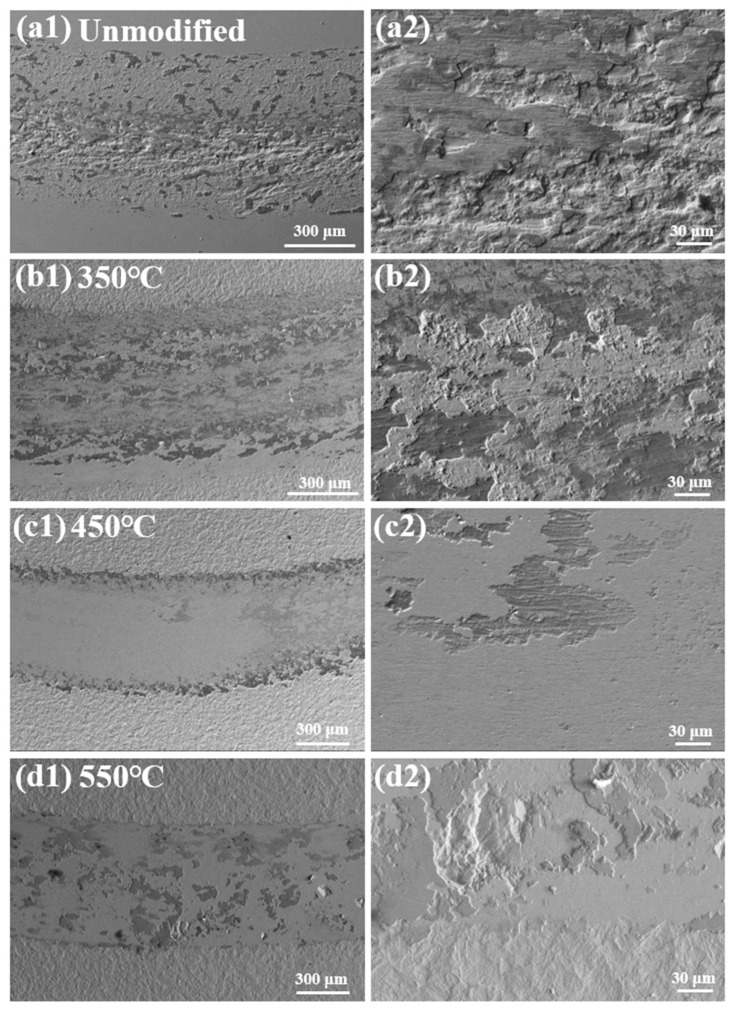
Worn surface morphologies of the unmodified and nitrided 17-4PH martensitic stainless steel at 350–550 °C against Si_3_N_4_ ball counterface under the sliding speed of 0.60 m/s and the normal load of 2 N. (**a1**,**a2**) Unmodified, (**b1**,**b2**) 350 °C, (**c1**,**c2**) 450 °C and (**d1**,**d2**) 550 °C.

Figure 10 shows the worn surface morphologies of the unmodified and nitrided 17-4PH martensitic stainless steel at 350–550 °C against Si_3_N_4_ ball counterface under the sliding speed of 0.60 m/s and the normal load of 2 N. When the sliding speed increases to 0.60 m/s, the wear track morphologies exhibit distinct deviations from that at the lower sliding speed of 0.15 m/s, directly reflecting the speed-dependent wear mechanism transitions. The unmodified martensitic stainless steel generates a rough wear track with deep directional furrows and layered spalling, as the severe adhesive wear governs the materials removal process. At 350 °C, the wear track displays fragmented oxide films and discontinuous flake-like spalls. The high-magnification imaging reveals the irregularly distributed shallow pits. Therefore, oxidative and fatigue wear jointly dominate the wear behaviors. Increasing to 450 °C, the worn surface retains a relatively smooth wear track covered by only faint oxide films. The oxidative wear still dominates at the higher sliding speed. At 550 °C, large cracked spalls and fragmented debris emerge on the worn surface with fewer oxide films. The fatigue wear and oxidative wear intensifies significantly under these conditions.

**Figure 11:**
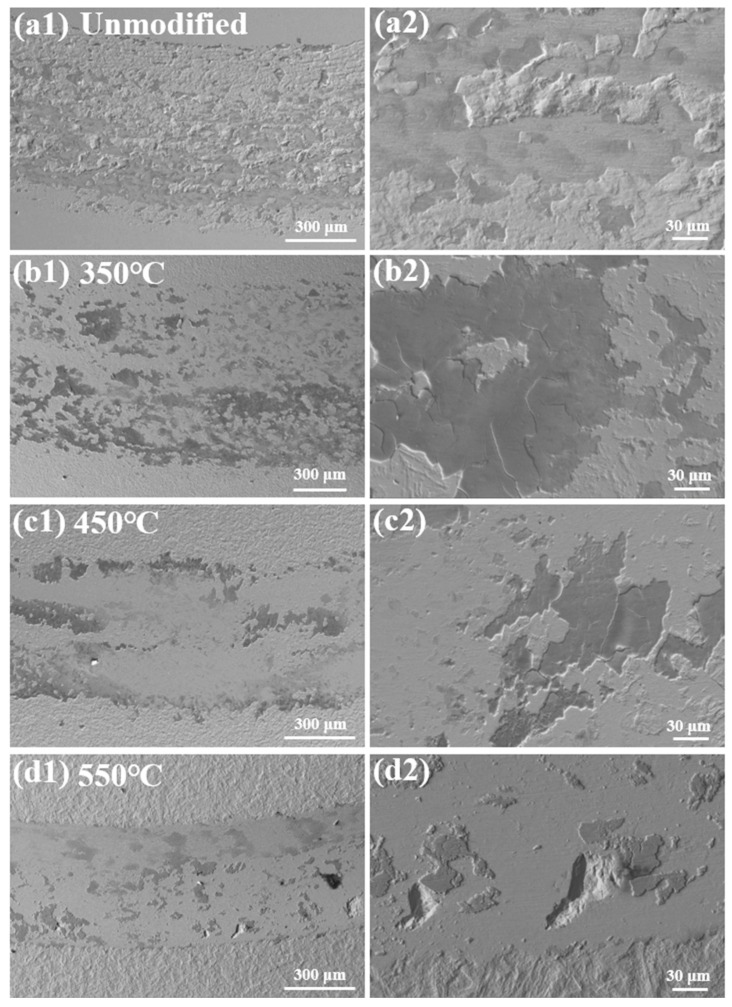
Worn surface morphologies of the unmodified and nitrided 17-4PH martensitic stainless steel at 350–550 °C against Si_3_N_4_ ball counterface under the sliding speed of 0.15 m/s and the normal load of 8 N. (**a1**,**a2**) Unmodified, (**b1**,**b2**) 350 °C, (**c1**,**c2**) 450 °C and (**d1**,**d2**) 550 °C.

Figure 11 shows the worn surface morphologies of the unmodified and nitrided 17-4PH martensitic stainless steel at 350–550 °C against Si_3_N_4_ ball counterface under the sliding speed of 0.15 m/s and the normal load of 8 N. When the normal load increases to 8 N and the sliding speed remains 0.15 m/s, the load-dependent wear mechanism transitions are distinctly observed by the corresponding changes in the wear track morphologies. For the unmodified martensitic stainless steel, extensive spalling, blocky debris and fewer oxide films cover the rough wear track. The wear behavior of the unmodified martensitic stainless steel is dominated by the adhesive and oxidative mechanisms. For the nitrided layer at 350 °C and 450 °C, the wear tracks both contain massive fragmented oxide films, indicating the oxidative wear. For the nitrided layer at 550 °C, large regular spalling pits appear on the worn surface covered by fewer oxide films. The wear mechanism transforms into the fatigue wear and minor oxidative wear with the sliding speed of 0.15 m/s and increasing the normal load to 8 N.

**Figure 12:**
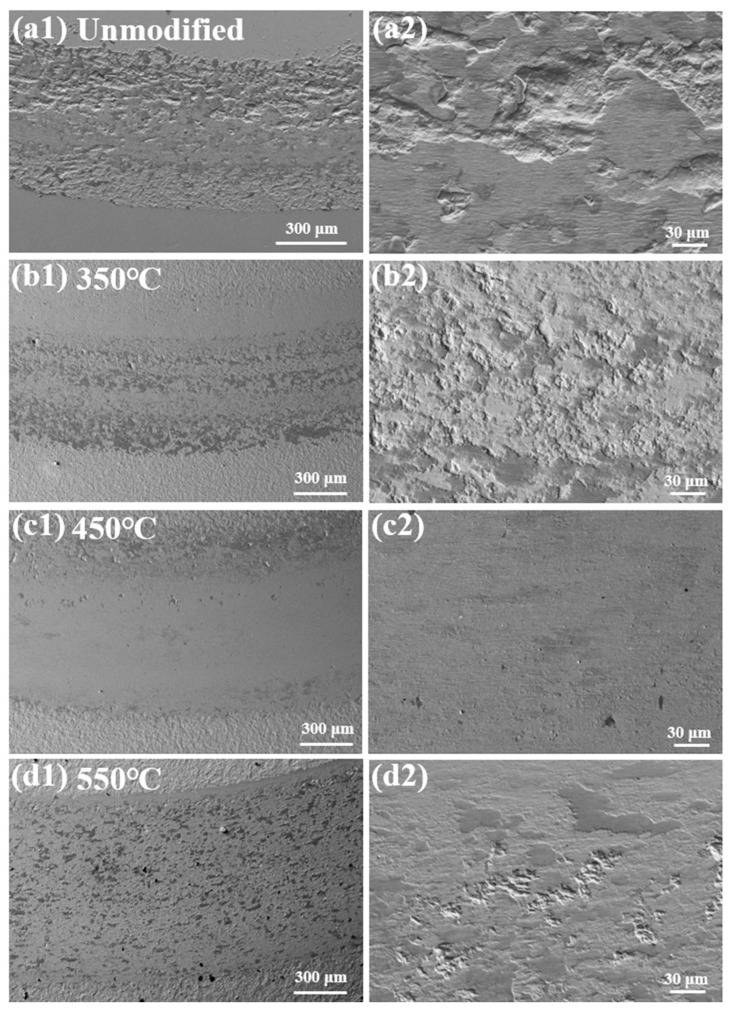
Worn surface morphologies of the unmodified and nitrided 17-4PH martensitic stainless steel at 350–550 °C against Si_3_N_4_ ball counterface under the sliding speed of 0.60 m/s and the normal load of 8 N. (**a1**,**a2**) Unmodified, (**b1**,**b2**) 350 °C, (**c1**,**c2**) 450 °C and (**d1**,**d2**) 550 °C.

Figure 12 shows the worn surface morphologies of the unmodified and nitrided 17-4PH martensitic stainless steel at 350–550 °C against Si_3_N_4_ ball counterface under the sliding speed of 0.60 m/s and the normal load of 8 N. A wear track with deep furrows and extensive spalling forms on the unmodified martensitic stainless steel under the severe wear condition. Meanwhile, fewer oxide films cover the wear track. The wear process is dominated by the severe adhesive wear coupled with the mild oxidative wear. At 350 °C, fragmented oxide films and fine spalls form on the wear track. The mixed oxidative and fatigue wear dominate the wear behaviors. Increasing to 450 °C, the worn surface shows the relatively smooth wear track with fewer oxide films, indicating the oxidative wear mechanism. At 550 °C, massive scattered pits and fragmented oxide films emerge on the worn surface. The fatigue wear intensifies significantly under the severe wear conditions of the normal load to 8 N and the sliding speed of 0.60 m/s, with the oxidative wear remaining minor.

**Figure 13:**
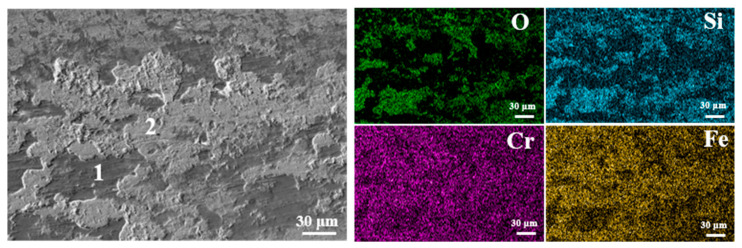
EDS mapping of surface wear track on the nitrided 17-4PH martensitic stainless steel at 350 °C against Si_3_N_4_ ball counterface under the sliding speed of 0.60 m/s and the normal load of 2 N. The quantitative EDS results at points 1 and 2 are presented in Table 2.

To further characterize the oxide film on the worn surface, elemental distribution mapping was employed to elucidate the wear mechanism. Figure 13 shows the EDS mapping of wear track on the nitrided 17-4PH martensitic stainless steel at 350 °C against Si_3_N_4_ ball counterface under the sliding speed of 0.60 m/s and the normal load of 2 N. The quantitative analysis results of the Point 1 and 2 were listed in Table 2 The elemental distribution on the worn surface demonstrates the formation of oxide films mainly consisting of Si, Cr and Fe. The Si element originated from the materials transfer of the Si_3_N_4_ ball counterface during sliding. The presence of Cr- and Fe-based oxides confirms the oxidative wear behaviors for the nitrided stainless steel. These oxide films also severe as a lubricating layer during sliding, effectively reducing the friction coefficient and the wear volume.

## 4. Discussion

The evolution of wear morphology and the corresponding wear mechanisms in 17-4PH martensitic stainless steel fundamentally depend on the microstructure and hardness of the nitrided layers. For the unmodified stainless steel, the relatively low surface hardness of approximately HV_0.25 N_ 3.05 GPa results in inadequate resistance to plastic deformation and material removal. This leads to a rough wear track characterized by furrows and spalled debris, with wear behavior dominated by a mild-to-severe adhesive mechanism. In contrast to the unmodified stainless steel, the nitrided layers exhibit significantly enhanced wear resistance against a Si_3_N_4_ ball counterface under all the sliding conditions. For the nintrided 17-4PH stainless steel, wear behavior under sliding is predominantly governed by an oxidative mechanism [9,12,21]. Nitriding at 350 °C for 4 h mainly produces nitrogen-expanded austenite γ’_N_ with little ε_N_ formed by stacking faults on the surface. When the temperature rises to 450 °C, CrN precipitates emerge and the volume fraction increases progressively with nitriding temperature up to 550 °C. Concurrently, the surface nitrogen concentration increases from approximately 29.7 at.% to 33.1 at.% for nitrided layers from 350 °C to 550 °C, while the corresponding surface hardness rises from 13.51 GPa to 15.66 GPa. These high-hardness nitrided layers significantly improve resistance to plastic deformation. The tribochemical oxidation of nitrogen-expanded austenite facilitates the formation of protective oxide films on the worn surface during sliding wear, similar to the behavior observed in nitrided austenitic stainless steel [22]. Furthermore, the nanostructure featuring a high density of grain boundaries within the nitrided layer accelerates oxide film formation. This oxide film effectively enhances wear resistance [23].

Under the various wear conditions, the surface of the nitrided layer at 450 °C exhibited the most outstanding wear resistance performance. For the light load conditions of 2 N and 0.15 m/s, the specific wear rate was approximately 0.58 × 10^−6^ mm^3^N^−1^m^−1^, indicating an extremely low material loss rate. Under the heavy load conditions of 8 N and 0.60 m/s, this value increased to 3.80 × 10^−6^ mm^3^N^−1^m^−1^, which remained markedly superior to nitrided layers at other temperatures. The excellent performance primarily stems from the significant differences in the microstructure of the nitrided layer at different temperatures: The nitrided layer formed at 350 °C is thinner and has lower surface hardness, making it prone to localized fatigue fracture and spalling during cyclic dry friction, leading to fatigue wear. Local intact regions on the worn surface show thinner oxide films, providing partial protection, so the wear mechanism at this temperature manifests as a composite pattern of oxidative wear and mild fatigue wear. Treatment at 450 °C forms a thicker and denser nitrided strengthening layer, whose high surface hardness mainly originates from the uniformly distributed nanocrystalline γ′_N_ phase and a small amount of dispersed CrN phase in the microstructure. This composite structure significantly enhances the material’s ability to resist elastic-plastic deformation during cyclic dry friction, effectively suppressing the initiation and propagation of fatigue cracks. The worn track surface exhibits only minor oxidation, which is presumed to be related to the rapid formation of protective oxide films promoted by the nanostructure. At 550 °C, the excessively high temperature leads to the extensive formation of high-volume fraction CrN precipitates, resulting in a decrease in the overall toughness of the nitrided layer. Cross-sectional microscopic observations clearly reveal multiple micro-cracks near surface in the grain boundaries in Figure 1. During cyclic dry sliding friction, these micro-cracks are more prone to nucleation, propagation, and penetration, ultimately leading to extensive layered delamination. At this stage, the wear mechanism shifts to a predominant fatigue wear pattern accompanied by minor oxidative wear, with specific wear rate increasing significantly, while the wear resistance performance markedly deteriorates.

## 5. Conclusions

(1) Nanostructured nitrided layers were synthesized on 17-4PH stainless steel via plasma-based low-energy nitrogen ion implantation at 350–550 °C for 4 h. Increasing nitriding temperature from 350 °C to 550 °C enhanced nitrided layer thickness from 11 μm to 27 μm and elevated surface nitrogen concentration from 29.7 at.% to 33.1 at.%. Concurrently, nanocrystalline size increased from 2 nm to 15 nm. The nitrided layer predominantly comprised γ′_N_ with minor ε_N_ at 350 °C. CrN precipitation occurred at 450 °C, facilitating complete transformation of α′_N_ to γ′_N_. At 550 °C, γ′_N_ decomposition yielded α_N_ and additional CrN precipitates.
(2) The surface hardness of the nitrided layers increases from 13.51 GPa to 15.66 GPa with the increasing nitriding temperature from 350 °C to 550 °C. All nitrided specimens demonstrated significantly enhanced wear resistance compared to the unmodified stainless steel. The wear mechanisms shifted from severe adhesive wear for the unmodified stainless steel to oxidative and/or fatigue wear for the nitrided specimens. The minimum wear rate was attained at a nitriding temperature of 450 °C.
(3) The layer treated at 450 °C exhibits lowest specific wear rates, attributable to its nanocrystalline γ’_N_ matrix and minor CrN precipitates. These microstructural characteristics synergistically enhance resistance to plastic deformation and facilitate protective oxide film formation. In contrast, the 350 °C treated layer demonstrates reduced thickness and lower surface hardness. Conversely, the higher nitriding temperature 550 °C induces excessive CrN precipitation and micro-cracking, which detrimentally affect both toughness and wear resistance.

## Figures and Tables

**Table 1:** Nitriding parameters for 17-4 PH martensitic stainless steel.

Microwave Power (W)	Base Pressure (Pa)	Nitriding Pressure (Pa)	Pulsed Negative Bias			Nitriding Temperature (°C)	Nitriding Time (h)
			Voltage (kV)	Repetition Rate (Hz)	Length (μs)		
200	1.5 × 10^−3^	5 × 10^−2^	−2	1000	250	350–550	4

**Table 2:** EDS results of surface wear track on the nitrided 17-4 PH martensitic stainless steel at 350 °C.

Content (at.%)	O	Si	Fe	Cr
Point 1	58.17	6.89	18.73	4.79
Point 2	20.11	1.88	36.06	8.79

## Data Availability

The original contributions presented in this study are included in the article. Further inquiries can be directed to the corresponding author.
